# Preterm birth and its associated factors among women of reproductive age in Zambia: a survey analysis

**DOI:** 10.1186/s12884-025-07808-5

**Published:** 2025-07-09

**Authors:** Mutale Sampa, Atupele Chisiza, Mwiche Musukuma, Ronald Fisa, Leah Kamulaza, Wilbroad Mutale

**Affiliations:** 1 School of Public Health, Department of Epidemiology and Biostatistics, The University of Zambia, Lusaka, Zambia; 2 School of Public Health, Department of Health Policy, and Management, The University of Zambia, Lusaka, Zambia; 3 Southern African Institute for Collaborative Research and Innovation Organization (SAICRIO), Lusaka, Zambia

**Keywords:** Preterm birth, Survey analysis, Reproductive, Prevalence

## Abstract

**Background:**

Preterm birth is the leading cause of death among children under five globally, with the highest burden in low- and middle-income countries (LMICs), particularly in Sub-Saharan Africa (SSA). Prevalence varies widely, ranging from 5% in high-income countries to 18% in LMICs. Despite its public health significance, Zambia lacks comprehensive nationally representative data on preterm birth. This study aimed to determine the prevalence and associated factors of preterm birth using data from the Zambia Demographic and Health Survey (ZDHS).

**Methods:**

The study analyzed data from the 2018 ZDHS, which used a two-stage stratified cluster sampling design. A total of 10,962 women aged 15–49 years who had a live birth within five years of the survey were included. Survey analysis techniques were used to account for the DHS complex sampling design. Stepwise survey logistic and log-linear regressions were fitted to identify factors associated with preterm birth. A p-value of < 0.05 was considered statistically significant.

**Results:**

The prevalence of preterm birth was 7%. Women with a parity of 10 or more had significantly increased prevalence of preterm birth (prevalence ratio (PR) = 2.15, 95% CI: 1.14–4.06, *p* = 0.018) compared to those with fewer than five births. Women who attended four or more antenatal care (ANC) visits had reduced prevalence of preterm birth (PR = 0.54, 95% CI: 0.46–0.65, *p* < 0.001). A history of abortion was also associated with higher odds of preterm birth (PR = 1.48, 95% CI: 1.14–1.93, *p* = 0.004).

**Conclusion:**

Preterm birth remains a critical public health concern in Zambia, with a prevalence of 7%, aligning closely with regional SSA estimates (~ 12% per WHO). Key risk factors include high parity, a history of abortion, and cesarean delivery, while older maternal age and frequent ANC visits are protective. These findings underscore the need to strengthen ANC services and implement targeted interventions for high-risk women to reduce the burden of preterm birth in Zambia.

## Background

Preterm birth (defined as birth before 37 completed gestation weeks of age [1], remains a significant public health challenge affecting both developed and developing countries. In 2020, an estimated 13.4 million babies were born preterm [2]. The preterm birth rates vary across regions, from as low as 5% in some developed countries to as high as 18% in LMICs, with the highest rates in SSA [3, 4].

Pre-term birth is associated with a higher risk of complications and co-morbidities, which affect both families and the health system. Globally, complications of pre-term births are a leading cause of childhood mortality, with low and middle-income countries (LMICs) disproportionately bearing this burden, particularly Sub-Saharan African countries (SSA) [5].

It is estimated that 1 million children die each year as a result of preterm birth complications [4]. The burden of preterm births places a significant strain on the health systems of SSA countries, requiring substantial investments in neonatal care facilities, specialized medical equipment, and trained healthcare professionals. Furthermore, the consequences of preterm birth may persist throughout their lives, impairing neuro-developmental functioning, impairing learning and vision, and compromising their long-term physical health [6–8]. Babies born preterm are prone to neonatal problems such as infection and respiratory complications, which may require longer hospital stay [9].

Several risk factors are known to contribute to Preterm birth. These include maternal age, parity, nutritional status, and infections during pregnancy [5, 10]. Factors associated with preterm birth include genetic, demographic, obstetrical and gynaecological, and other medical factors [11]. The distribution of these risk factors varies across countries and are context-specific [11, 12]. In SSA, where the rate of adolescent pregnancy is high, there is a higher risk for PTB [13]. In low-income settings, such as in SSA, there is a strong association between preterm birth and infection, including Human immunodeficiency virus (HIV) infection, due to the high burden of infection in these settings [3, 14–16].

In Zambia, the preterm birth rate is estimated to be 13%, and each year 77,600 preterm births and 6,800 infant deaths are due to preterm birth complications [17]. This high prevalence of preterm births is attributed to various factors, including inadequate access to quality antenatal care, limited availability of skilled birth attendants, and socio-economic disparities. Systemic challenges, including inadequate healthcare infrastructure and resource constraints have hindered efforts to address preterm birth in Zambia [10]. Despite these challenges, initiatives such as the Saving Mothers, Giving Life (SMGL) program have shown promising results in reducing maternal and neonatal mortality rates, including preterm births, through targeted interventions in Zambia. However, preterm birth remains a persistent public health challenge in Zambia, necessitating comprehensive strategies to address its multifaceted determinants.

Preterm birth is a significant contributor to infant mortality and long-term health issues globally. In Zambia, understanding the prevalence and determinants of preterm birth is crucial for developing effective public health strategies. Despite its importance, there is a lack of comprehensive nationally represented data on preterm birth in Zambia, particularly regarding socioeconomic factors and provincial disparities. Primary data would be ideal for getting comprehensive risk factors of preterm birth in Zambia. However, Zambia being a LMIC, it has limited resources to collect extensive primary data on preterm birth countrywide.

While the ZDHS primarily captures distal and socio-demographic determinants, it offers a unique opportunity to examine population-level trends and inequalities in preterm birth across provinces in Zambia. The ZDHS added a variable which records the duration of pregnancy in months, this can be used as a proxy for gestational age. As a nationally representative dataset, the ZDHS provides detailed information on maternal and child health indicators, including preterm birth, across different socioeconomic groups and regions. The use of ZDHS data ensures that findings will be generalizable and relevant for national health policy development. By analyzing ZDHS data, this study aims to determine prevalence and factors associated with preterm birth. This research is significant because it will provide policymakers and healthcare providers with valuable insights to inform targeted interventions aimed at reducing preterm birth rates and improving maternal and child health outcomes in Zambia.

## Methodology

### Study design and data source

To estimate the prevalence of preterm births and associated factors in Zambia, we used data from the 2018 Zambia Demographic and Health Survey (ZDHS). The ZDHS survey data is collected using structured, pre-tested, and validated questionnaires every five years. The survey also follows standardized procedures in developing questionnaires, sampling, data collection, and coding.

### Sampling, study population, and sample size

A two-stage stratified cluster sampling technique was employed. The first stage was sampling enumeration areas (EAs), which are also considered clusters, and the second stage was sampling households listed in each cluster. The population of interest in this study was women of reproductive age (15–49 years), and a total sample of 10,962 women who had given birth in the five years preceding the survey was included. Women with missing values of the duration of pregnancy were excluded from the study as this was the variable used to generate the outcome variable.

### Study variables

The outcome variable for this study was preterm birth among women of reproductive age. This variable was generated from the ZDHS question, “duration of pregnancy (b20 from the birth recode file),” which was dichotomized as “Yes” for preterm birth for births that were delivered eight completed months of gestational age or before, and “No” if the birth happened nine or more completed months of gestational age. The most recent birth was used for women who had birth more than once within five years preceding the survey.

This study’s independent variables included demographic, socioeconomic, and health-related factors. The variables included women’s age (in five year age group), education level (no education, primary, secondary, higher) [18], marital status (never in union, married, living with partner, widowed, divorced, no longer living together/separated), wealth quintile (poorest, poorer, middle, richer, richest) [7], currently employed(yes or no), number of Antenatal Care (ANC) visits this was categorized in to three categories (no ANC visits, < four and ≥ four visits) was classified as optimal if a mother had more than 4 visits during her pregnancy and non-optimal if the mother had less than 4 ANC visits during her pregnancy [18], history of terminated pregnancy (no or yes), previous delivery by cesarean section (no or yes) were included as the individual level variables of the study. Residence (urban and rural) and province were included as the community-level variables. Similar studies by Tebeka et al. 2024, and Alamneh et al., 2021 which analyzed Demographic Health Survey (DHS) data for SSA included similar independent variables in the analysis [7, 18].

### Statistical analysis

All analyses used STATA^®^ version 17 (StataCorp, College Station, TX, USA) software. Data was cleaned, and this study applied survey analysis to account for the sampling design in the ZDHS. The survey analysis accounted for clusters, stratification and the weights in the DHS design. Before any analysis could be done, data was set for survey analysis to enable using survey commands in Stata. The following syntax was used.

### Svyset cluster [pweight = weight], strata(strata) Vce (linearized)

All commands in Stata included a svy in front indicating survey analysis. Descriptive statistics were done, reporting frequencies and proportions (for all categorical variables). The chi-square test was used to check for any association between preterm birth and other explanatory variables.

To estimate factors associated with preterm birth in Zambia, two models were fitted an investigator-led stepwise survey logistic regression and survey log-linear regression with a Poisson model using Generalized Estimating Equations (GLM), which is well-suited for cross-sectional data with binary outcomes where the event is not rare [19]. Odds ratios were reported for the logistic regression and for the log-linear regression, prevalence ratios (PR) with corresponding confidence intervals were reported.

The Pearson r Homsmer-Lemeshow goodness-of-fit test was used to determine the best-fit model; a smaller chi-square value with a large P-value closer to one indicated a good model fit. A model without education, Residence, Wealth quintile, Employment, distance, and ANC in 1st Trimester (Chi-square 0.76, P-value 0.6556).

## Results

Table 1 below shows descriptive results and the chi-square test results, which assessed the association between the preterm birth and various explanatory variables.

**Table 1:** Background characteristics of women of reproductive age and association of preterm birth and explanatory variables

Variable	Total *N* = 10,962 (%)	Full term Birth *N* = 10,161 (%)	Preterm Birth *N* = 801 (%)	*P*-Value
Marital Status Never in union Married Living with partner Widowed Divorced No longer living together/separated	1186 (10.8) 8492 (77.5) 41(0.4) 165 (1.5) 706 (6.4) 372 (3.4)	1095 (10.8) 7891 (77.7) 39 (0.4) 148 (1.5) 652 (6.4) 336 (3.3)	91 (11.4) 601 (75.0) 2 (0.2) 17 (2.1) 54 (6.7) 36 (4.5)	0.248
Educational status No education primary secondary higher		1126 (11.1) 5373 (52.9) 3270 (32.2) 392 (3.9)	79 (9.9) 387 (48.3) 299 (37.3) 36 (4.5)	
	1205 (11.0) 5760 (52.5) 3569 (32.6) 428 (3.9)			0.013
**Age in 5-year groups** 15–19 20–24 25–29 30–34 35–39 40–44 45–49	836 (7.6) 2834 (25.8) 2643 (24.1) 2172 (19.8) 1508 (13.7) 774 (7.1) 195 (1.8)	752 (7.4) 2597 (25.6) 2454 (24.1) 2024 (19.9) 1416 (13.9) 734 (7.2) 184 (1.8)	84 (10.5) 237 (29.6) 189 (23.6) 148 (18.5) 92 (11.5) 40 (5.0) 11 (1.4)	0.001
**Currently Employed** No Yes	5698 (52.0) 5264 (48.0)	5257 (51.7) 4904 (48.3)	441 (55.1) 360 (44.9)	0.070
**Distance to the health facility** Big problem Not a big problem	4069 (37.1) 6893 (62.9)	3756 (37.0) 6405 (63.0)	313 (39.1) 488 (60.9)	0.234
**Number of ANC Visits** No ANC visits Less than 4 4 or more	89 (1.2) 2476 (33.6) 4807 (65.2)	79 (1.2) 2222 (32.5) 4537 (66.3)	10 (1.9) 254 (47.6) 270 (50.6)	< 0.001
**Parity** Less than Five Five to Nine Ten or more	7296 (66.6) 3388 (30.9) 278 (2.5)	6722 (66.1) 3186 (31.4) 253 (2.5)	574 (71.7) 202 (25.2) 25 (3.1)	0.001
**Previous delivery by cesarean section** No Yes	10,452 (95.4) 510 (4.6)	9698 (95.4) 463 (4.6)	754 (94.1) 47 (5.9)	0.090
**History of a terminated pregnancy** No Yes	9961 (90.9) 1001 (9.1)	9256 (91.1) 905 (8.9)	705 (88.0) 96 (12.0)	0.004
**ANC in the first trimester** No Yes	4521 (62.1) 2762 (37.9)	4185 (61.9) 2574 (38.1)	336 (64.1) 188 (35.9)	0.316
**Wealth Index** Poorest Poorer Middle Richer Richest	3160 (28.8) 2647 (24.1) 2159 (19.7) 1597 (14.6) 1399 (12.8)	2923 (28.8) 2473 (24.3) 20,001 (19.7) 14,477 (14.5) 1287 (12.7)	237 (29.6) 174 (21.7) 158 (19.7) 120 (15.0) 112 (14.0)	0.495
**Region** Urban Rural	3238 (29.5) 7724 (70.5)	2981 (29.3) 7180 (70.7)	257 (32.1) 544 (67.9)	0.101
**Province** Central Copperbelt Eastern Luapula Lusaka Muchinga Northern Northwestern Southern Western	1083 (9.9) 1007 (9.2) 1308 (11.9) 1348 (12.3) 1134 (10.3) 1028 (9.4) 1119 (10.2) 890 (8.1) 1125 (10.3) 920 (8.4)	935 (9.2) 938 (9.2) 1218 (12.0) 1256 (12.4) 1055 (10.4) 949 (9.3) 1042 (10.3) 855 (8.4) 1075 (10.6) 838 (8.2)	148 (18.5) 69 (8.6) 90 (11.2) 92 (11.5) 79 (9.7) 79 (9.9) 77 (9.6) 35 (4.4) 50 (6.24) 82 (10.2)	< 0.001

### Demographic characteristics of women

The study included approximately 10,900 women of reproductive age, as shown in Table 1 below. The majority of the 8492 (77%) women reported being married, and about 50% had gone up to the primary school level. The majority of these women were from rural areas; 7724 (70%) and 3160 (29%) were in the poorest wealth index bracket. Over 505 of the women were between the ages of 20 to 34 years.

The prevalence of preterm birth was estimated at 7%; 801 women out of the total women included in the study had delivered their babies prior to 8 completed of months of gestation period.

### Bivariate analysis of preterm birth

The results from the Chi-square test in Table 1 found the following variables to be associated with preterm birth: age, level of education, province where the woman was coming from, parity (the number of children a woman has ever had), a woman’s history of a terminated pregnancy, and the number of ANC visits a woman attended. These variables were found to be associated with preterm births with a P-value of less than 0.05.

**Fig. 1:**
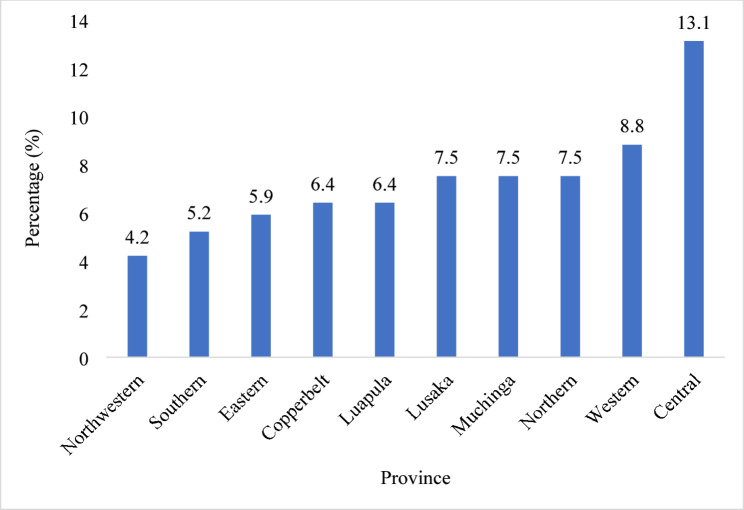
Prevalence of preterm birth in Zambia by province

Figure 1 shows the prevalence of preterm birth by province. The highest prevalence was observed in Central province with 13.1%, followed by Western province with 8.8%. Lusaka, Muchinga and Northern provinces had a prevalence of 7.5%. The lowest prevalence of PTB was in Northwestern province with 4.2%.

**Fig. 2:**
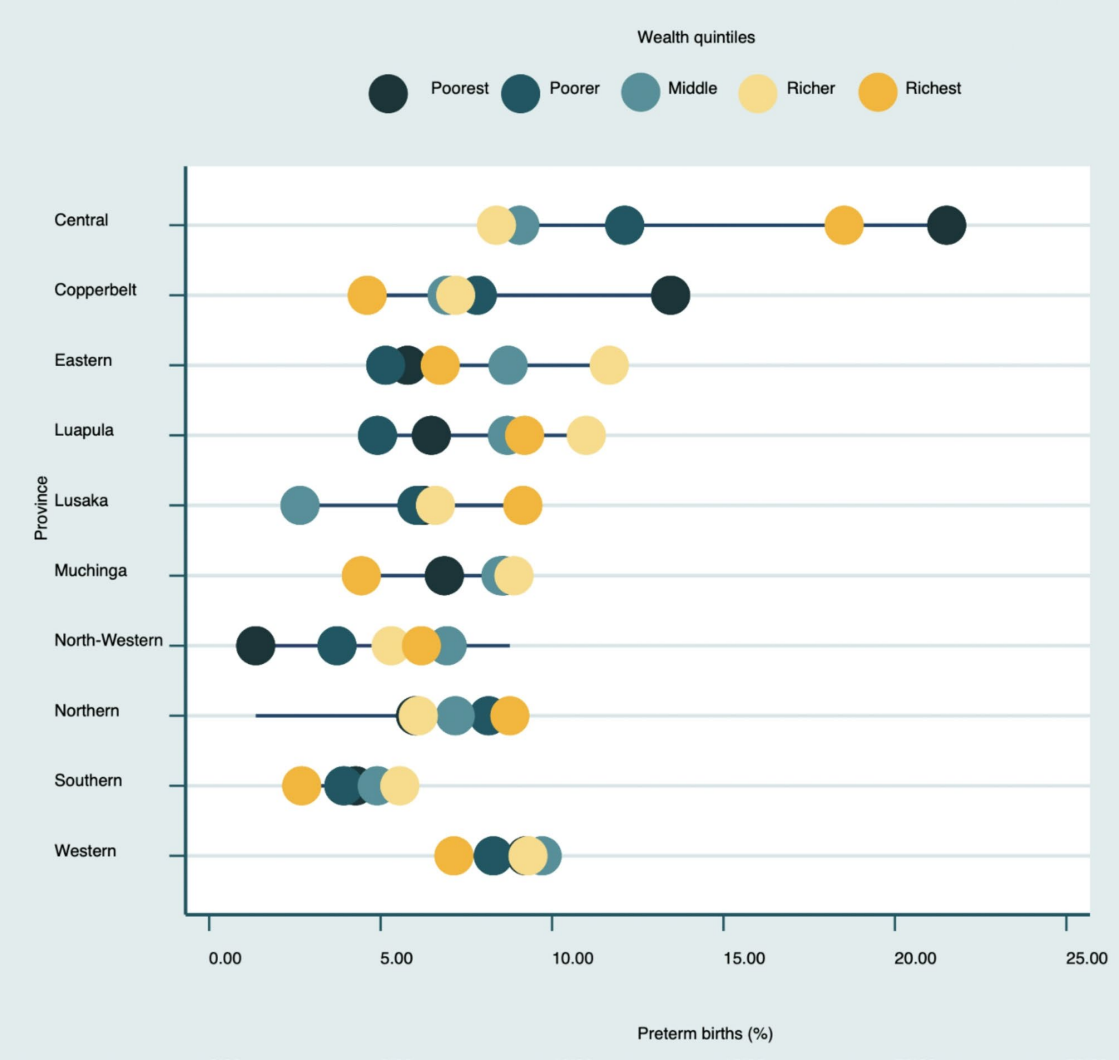
Equity of plot of preterm birth by province and wealth quintile

Figure 2 shows the equity plot of preterm birth by province and wealth quintile. The plot shows the inequalities in the burden of preterm birth in the provinces by wealth quintile. The largest gap being observed in central province where women in the poorest wealth quintile had a prevalence of preterm birth of 21.5%, followed by women in the richest wealth quintile about 18.5% and the lowest in the province being the rich with 8.4%. In Copperbelt province women in the poorest wealth quintile had the highest prevalence of about 13.5%. In Lusaka however, the highest prevalence was observed among the richest of 9.2%.

### Factors associated with preterm births

Table 2 shows the results from the multivariable survey logistic regression and the log-linear regression analysis. The two models were fitted to compare the estimates. Slight differences were observed, and therefore, the study interpreted the results from the log-linear regression model since the preterm birth is not a rare outcome.

**Table 2:** Multivariable analysis of factors associated with preterm birth among reproductive age women in Zambia

Variable	Model 1		Model 2	
	Odds Ratio (95% CI)	*p*-value	Prevalence Ratio (95% CI)	*p*-value
Age in 5-year groups 15–19 20–24 25–29 30–34 35–39 40–44 45–49	Reference 0.69 (0.41–1.14) 0.65 (0.43–0.99) 0.59 (0.35–0.99) 0.54 (0.29–1.01) 0.36 (0.18–0.70) 0.37 (0.15–0.94)	0.146 0.048 0.048 0.054 0.003 0.037	Reference 0.71 (0.46–1.12) 0.68 (0.47–0.99) 0.62 (0.39–0.99) 0.58 (0.33–1.01) 0.39 (0.21–0.72) 0.41 (0.17–0.95)	0.142 0.044 0.046 0.053 0.003 0.038
**Marital Status** Never in union Married living with partner widowed divorced No longer living together/separated	Reference 1.17 (0.79–1.73) 1.31 (0.28–6.23) 2.31 (0.94–5.68) 2.20 (1.07–4.52) 1.97 (1.08–3.57)	0.425 0.730 0.068 0.032 0.027	Reference 1.15 (0.81–1.64) 1.28 (0.31–5.29) 2.12 (0.96–4.68) 2.01 (1.07–3.78) 1.82 (1.08–3.06)	0.432 0.731 0.063 0.029 0.025
**Parity** Less than 5 5 to 9 10 or more	Reference 0.81 (0.58–1.13) 2.34 (1.13–4.86)	0.213 0.022	Reference 0.82 (0.61–1.12) 2.15 (1.14–4.06)	0.214 0.018
**Previous delivery by cesarean section** No Yes	Reference 1.46 (0.93–2.28)	0.097	Reference 1.40 (0.94–2.07)	0.097
**Number of ANC Visits** Less than 4 4 or more	Reference 0.51 (0.420–0.63)	< 0.001	Reference 0.54 (0.46–0.65)	< 0.001
**History of a terminated Pregnancy.** No Yes	Reference 1.55 (1.15–2.10)	0.005	Reference 1.48 (1.14–1.93)	0.004
**Province** Central Copperbelt Eastern Luapula Lusaka Muchinga Northern Northwestern Southern Western	Reference 0.44 (0.27–0.71) 0.43 (0.27–0.69) 0.57 (0.38–0.86) 0.50 (0.32–0.79) 0.62 (0.38–1.00) 0.51 (0.31–0.83) 0.31 (0.19–0.50) 0.44 (0.23–0.83) 0.61 (0.39–0.97)	0.001 < 0.001 0.008 0.003 0.050 0.006 < 0.001 0.012 0.035	Reference 0.48 (0.31–0.74) 0.47 (0.32–0.72) 0.62 (0.43–0.88) 0.55 (0.32–0.79) 0.66 (0.43–1.00) 0.55 (0.36–0.85) 0.35 (0.22–0.54) 0.48 (0.27–0.86) 0.65 (0.44–0.97)	0.001 < 0.001 0.007 0.003 0.051 0.007 < 0.001 0.014 0.035

Model 1 = logistic regression, Model 2 = Log-Linear regression

Odd ratios and prevalence ratios were adjusted for other covariates listed in the table

The study found that women who were married, living with a partner, widowed, divorced, and No longer living together/separated had a higher prevalence of preterm birth compared to women who were never in the union, accounting for all other variables in the model. However, only the results for divorced and No longer living together/separated were statistically significant, with P-values of 0.032 and 0.027, respectively.

Accounting for all the other variables in the model, women in the age groups 20–24, 25–29, 30–34, 35–39, 40–44, and 45–49 had lower prevalence of preterm birth compared to women in the age group 15–19. For the age group 20–24, the results were not statistically significant (P-value = 0.142), while for the age group 35–39, the result was borderline with a P-value = 0.053. The rest of the age groups were found to be statistically significant with a P-value < 0.05.

Women with five to nine children compared to women with less than five children had a lower prevalence of preterm birth, but this was not statistically significant (PR = 0.82, CI = 0.61–1.12, P-value = 0.214). However, women with ten or more children compared to women with less than five children had a higher prevalence of preterm birth, and this was statistically significant (PR = 2.15, CI = 1.14–4.06, P-value = 0.018).

Women whose previous delivery was cesarean section had a higher prevalence preterm birth compared to women whose last delivery was a normal birth; this was not statistically significant (PR = 1.40, CI = 0.9–2.07, P-value = 0.097). Women who attended four or more ANC visits had a lower prevalence of preterm birth compared to women who attended less than four ANC visits, (PR = 0.54, CI = 0.46–0.65, P-value < 0.001). Women with a history of terminated pregnancy had a higher prevalence of preterm birth compared to those without a history, this was statistically significant (PR = 1.48, CI = 1.14–1.93, P-value = 0.004).

The prevalence of preterm birth was lower for women who lived in the following provinces: Copperbelt, Eastern, Luapula, Lusaka, Muchinga, Northern, Northwestern, Southern, and Western compared to women who lived in Central province. These findings were statistically significant except Muchinga province with a borderline significance P-value 0.051.

## Discussion

This study aimed to determine the prevalence and factors associated with preterm birth in Zambia using the ZDHS. The estimated prevalence of preterm birth in this study was 7.3%, which is lower than the estimated average prevalence in LMIC countries of 12% 9.4% and 9.3% for middle and high-income countries, respectively [2, 20]. Country-specific studies conducted in some low-income countries like Zambia Ethiopia and Uganda found a prevalence of 7.7%,11.4%and 13.6%, respectively [21–23]. Another study done in Kenya found the prevalence of preterm birth to be 18.7% at Kenyatta Hospital. In SSA the prevalence of preterm birth was 5.3% [7].

Our study also suggests that older women have lower prevalence of preterm birth compared to teenagers. This was consistent with a systematic review which found that women less than 20 years of age had increased odds of preterm birth [24]. However, contrary to this study’s finding, other studies conducted in Malawi and Kenya found that older mothers over 40 years had increased odds of having a preterm birth and women less than 20 years old had reduced odds of preterm birth respectively [25, 26]. The findings in our study indicate that teenagers are at a higher risk of preterm birth. This could be because teenage pregnancies are frequently unexpected, which can decrease the chances of young mothers pursuing proper prenatal and antenatal care. Younger women are also more likely to participate in risky behaviors, such as substance use, and may be less responsive to guidance and education from healthcare providers when compared to older women. Additionally, many teenage girls are not physically developed enough for pregnancy and childbirth, heightening the risk of preterm birth [7].

This study found that women who were divorced or separated had significant increased prevalence of preterm birth compared to women who have never been married or lived with a partner. This in in contrast with previous studies [7, 18, 23]. This might be related to potential high psychosocial stress among divorced or separated women due to pressure from the society. However, single women are also discriminated against when they get pregnant before marriage.

Our study findings suggest that a woman with a history of terminated pregnancy had a higher prevalence of preterm birth, and this is consistent with findings from a systematic review conducted in Ethiopia in which Women who had a history of abortion had a twofold increased risk of preterm birth [21]. If a woman had surgical abortion, particularly methods involving cervical dilation, their cervix can weaken or get damaged, increasing the risk of cervical incompetence which is a leading contributor to preterm labor, as the cervix may fail to remain closed during pregnancy [27].

This study found that women attending four or more ANC a lower prevalence of preterm birth, and this finding was consistent with the findings from a systematic review, which found that attending less than four ANCs increased the odds of preterm birth [24]. This is because they receive early risk mitigation such as hypertension detection and management, detection and treatment infections such as malaria. During ANC women receive behavioral and preventive counseling on several topic such as nutrition, and substance use which are contributors of preterm birth. Women with frequent ANC visits are likely to receive timely interventions for preterm birth prevention [28].

In this study, women with a parity of 10 children or more significantly had a higher prevalence of preterm birth. Similar to our findings, a study conducted at Kenyatta Hospital found that Women with a parity of 4 or more were nearly five times more likely to deliver preterm than those with less than four [25]. However, a study done in Zambia found that parity was not associated with preterm birth [23]. Repeated pregnancies and deliveries progressively weaken cervical integrity, increasing susceptibility to cervical insufficiency and preterm labor [29].

This study had some limitations; the analysis of preterm birth using ZDHS data relied on pregnancy duration reported in months, which introduced methodological limitations. While DHS surveys categorize gestational age as 5 to10 months, this approach lacks the precision of week-based definitions (< 37 completed weeks) as defined by WHO, potentially misclassifying borderline cases such as infants born at 36 weeks vs. 37 weeks. The 8 months duration might include both preterm and term births. This imprecision may have overestimated or underestimated the preterm birth prevalence and the prevalence ratios. Additionally, DHS’s reliance on women to recall pregnancy duration. This introduces recall bias, as women may inaccurately estimate gestational age, especially in settings with limited prenatal care access. These limitations highlight the need for standardized weeks-based gestational age reporting in future surveys to improve comparability. The study did not account for other important factors such as infections and substance use.

The other limitation for this study was that our analysis using DHS data introduced methodological limitations such as the sampling method when comparing findings to non-DHS studies. DHS surveys rely on maternal recall for gestational age and employ clustered sampling, which may introduce recall bias and design effects. On the other hand, non-DHS comparators are usually facility-based studies and often use alternative methodologies, leading to heterogeneity in study bases and risk estimates. facility-based studies may overrepresent high-risk populations such as the urban areas, while DHS reflects national-level trends. These differences risk misalignment in interpreting associations, particularly for outcomes like preterm birth, where the definition (months vs. weeks) and data details vary across studies.

While ZDHS provides critical insights into risk factors for preterm birth in Zambia, its reliance on month-based gestational age reporting and maternal recall limits its utility for precise prevalence estimation. Facility-based studies and population surveys using weeks-based definitions may yield more accurate rates, though ZDHS remains valuable for identifying modifiable determinants.

## Conclusion

This study found a preterm birth prevalence of 7.3% in Zambia. Key risk factors included a history of abortion, high parity, younger maternal age, and being formerly in union, while attending four or more ANC visits was protective. These findings highlight the need to strengthen antenatal care services, enhance support for younger and formerly married women, and promote family planning education to reduce preterm births in Zambia. While this study provides valuable insights, future research should aim to collect more precise gestational age data and include additional variables such as maternal smoking and alcohol consumption to understand further the factors influencing preterm birth.

## Data Availability

The dataset supporting this article’s findings can be obtained through the Demographic Health Survey program; however, other study materials can be provided upon reasonable request from the corresponding author, whose email is mutalesampa65@gmail.com.

## Abbreviations

LMICs: Low-and middle-income countries
SSA: Sub-Saharan African countries
ZDHS: Zambia Demographic and Health Survey
HIV: Human immunodeficiency virus
SMGL: Saving Mothers Giving Life
EAs: Enumeration areas
ANC: Antenatal Care
